# Population‐based strategies for dementia risk reduction in LMICs: Perspectives, Potentials and Priorities

**DOI:** 10.1002/alz70860_102285

**Published:** 2025-12-23

**Authors:** Rufus O. Akinyemi, Oludotun V Olalusi, Tobi Nifemi Olajide

**Affiliations:** ^1^ College of Medicine, University of Ibadan, Ibadan, Oyo State, Nigeria; ^2^ University College Hospital, Ibadan, Oyo, Nigeria; ^3^ College Research and Innovation Hub, Ibadan, Nigeria; ^4^ College of Medicine University of Ibadan, Ibadan, Nigeria

## Abstract

**Background:**

Globally, dementia risk reduction remains a significant public health priority. Existing and ongoing primary prevention strategies are focused largely on individual‐level interventions targeted at high‐risk individuals, with a wide research and policy gap for population‐level interventions. We conducted a scoping review to identify empirical evidence on population‐level interventions in LMICs for dementia and its modifiable risk factors.

**Methods:**

We searched PubMed and google scholar for relevant studies using pre‐determined keywords. Inclusion criteria were RCTs/quasi‐experimental studies and systematic reviews in LMICs. Outcomes included dementia incidence, cognitive decline, dementia‐specific risk factors and other traditional non‐communicable diseases (NCD) risk factors. The key strategies of interest include public health initiatives such as media campaigns, legislative changes, and community programs promoting healthier lifestyles (dietary, smoking and alcohol). Study quality was rated using relevant criteria. The review was summarized in three stages: i) identification of population‐level interventions for ‘traditional NCD’ risk factors in LMICs; (ii) identification of population‐level interventions for ‘dementia‐specific’ risk factors in LMICs; and (iii) review of population‐level dementia risk reduction in LMICs.

**Results:**

Following a comprehensive series of targeted searches, we identified 33 articles detailing interventions on smoking restriction, alcohol restriction, obesity reduction (via dietary interventions and taxes on sugar‐sweetened beverages), reduction in hypertension (salt restriction), physical inactivity (using mass media campaigns), air pollution, and low education. Specific/ongoing research efforts/priorities include the ACHIEVE study in Nigeria (tackling hypertension), Food‐EPI study in Guatemala (tackling obesity), African‐PREDICT study in South Africa, BRIGHT school construction program in Burkina Faso and Peruvian 2005 Juntos Conditional Cash Transfer (tackling low education), and Justa Stove in Honduras (tackling Air pollution). None of the articles had dementia/cognitive impairment as an outcome measure.

**Conclusion:**

The lessons from this review can be used to design context‐specific, tailored, population‐based interventions trials for dementia in LMICs. Given the relative successes of population‐based interventions on reducing the prevalence of NCDs and vascular risk factors, we propose that suitable integrated care models that target overlapping risk factors for dementia, cardiovascular disease/risks, and other non‐communicable diseases will provide a more holistic and scalable solution, particularly in LMICs.

